# A Systematic Review of Suicide Patterns in the Gulf Cooperation Council Countries

**DOI:** 10.7759/cureus.102564

**Published:** 2026-01-29

**Authors:** Sadiq F Alherz, Komail M Alramadhan, Abdullah Y Alramadan, Ahmed A Alsafwani, Naser K Alghadhban, Ritesh G Menezes

**Affiliations:** 1 College of Medicine, Imam Abdulrahman Bin Faisal University, Dammam, SAU

**Keywords:** arabian gulf region, expatriates, gcc countries, suicide, suicidology

## Abstract

Research evaluating suicide in Gulf Cooperation Council (GCC) countries remains limited in the literature. This systematic review aims to analyse existing literature on suicide in GCC countries with an emphasis on the demographic characteristics, common methods employed, and the risk factors contributing to suicide. A search for the relevant literature was performed using PubMed and the Web of Science Core Collection databases. Fifteen studies met the inclusion criteria. The findings consistently demonstrated a predominance of male cases. Expatriates, particularly individuals of Indian origin, represented the majority of cases. Suicide was more frequent among younger adults. Hanging emerged as the most frequent method, followed by jumping from a height. Additional research is required to assess the scope of the issue accurately. The implementation of targeted policies and preventive strategies is recommended, particularly for high-risk groups, such as expatriates.

## Introduction and background

Suicide is defined as the termination of life resulting from self-inflicted injury with the intent of dying [1-5]. The World Health Organization (WHO) reports that more than 720,000 individuals die by suicide annually, with many more attempting suicide [6]. Suicide represents a significant loss at the individual level. It also has adverse impacts on families, communities, and society. The aetiology of suicide remains complex and not fully elucidated [7,8]. It is, however, widely acknowledged as a multifactorial phenomenon influenced by social, biological, cultural, psychological, and environmental determinants [6-9].

Suicide rates in Gulf Cooperative Council (GCC) countries (Arabian Gulf region/Gulf region/Gulf countries) are substantially lower than the global average [10]. It is reported that in the Arab world (which includes GCC countries), suicide rates are lower when compared to the global average [10]. Nevertheless, suicide remains a public health concern in the Gulf region, likely underreported and underestimated. Mental health and suicide are frequently considered taboo, leading families to conceal suicide or attribute it to other categories of death. Cultural and religious factors, particularly those associated with Islam, contribute to the stigma surrounding suicide [10,11]. Comprehensive studies addressing all aspects of suicide in the Gulf region are limited.

This systematic review aims to synthesize existing evidence on suicide in Gulf countries, with particular emphasis on demographic characteristics, methods employed, and risk factors. By examining these components, the review seeks to provide insights that may inform future research directions and policy development in the region.

## Review

Methods

The eligibility criteria for this systematic review included English-language primary research articles that reported suicides in GCC countries regardless of age and gender. For a study to be eligible, it had to focus on any one of the GCC countries (Bahrain, Kuwait, Oman, Qatar, Saudi Arabia, and the United Arab Emirates) and include information such as demographic data, suicide methods, and risk factors. Studies focusing on specific subpopulations (e.g., individuals with schizophrenia or other psychiatric disorders) were excluded. Additionally, review articles, case reports, and case series were excluded. The primary search was conducted on October 11, 2024, using PubMed and Web of Science Core Collection databases. "Advanced search" and "all fields" were considered during the search on Web of Science Core Collection (all editions). The search strategy focused on the key terms related to "suicide" and "Gulf Cooperation Council (GCC) countries" (Table 1).

**Table 1:** Search strategy

Applied search terms	Database	Search results
(Suicide OR Suicidal) AND (GCC OR Gulf Cooperation Council OR Saudi OR KSA OR Kuwait OR Oman OR Bahrain OR Qatar OR United Arab Emirates OR UAE)	PubMed	545 records
	Web of Science Core Collection	843 records

**Figure 1:**
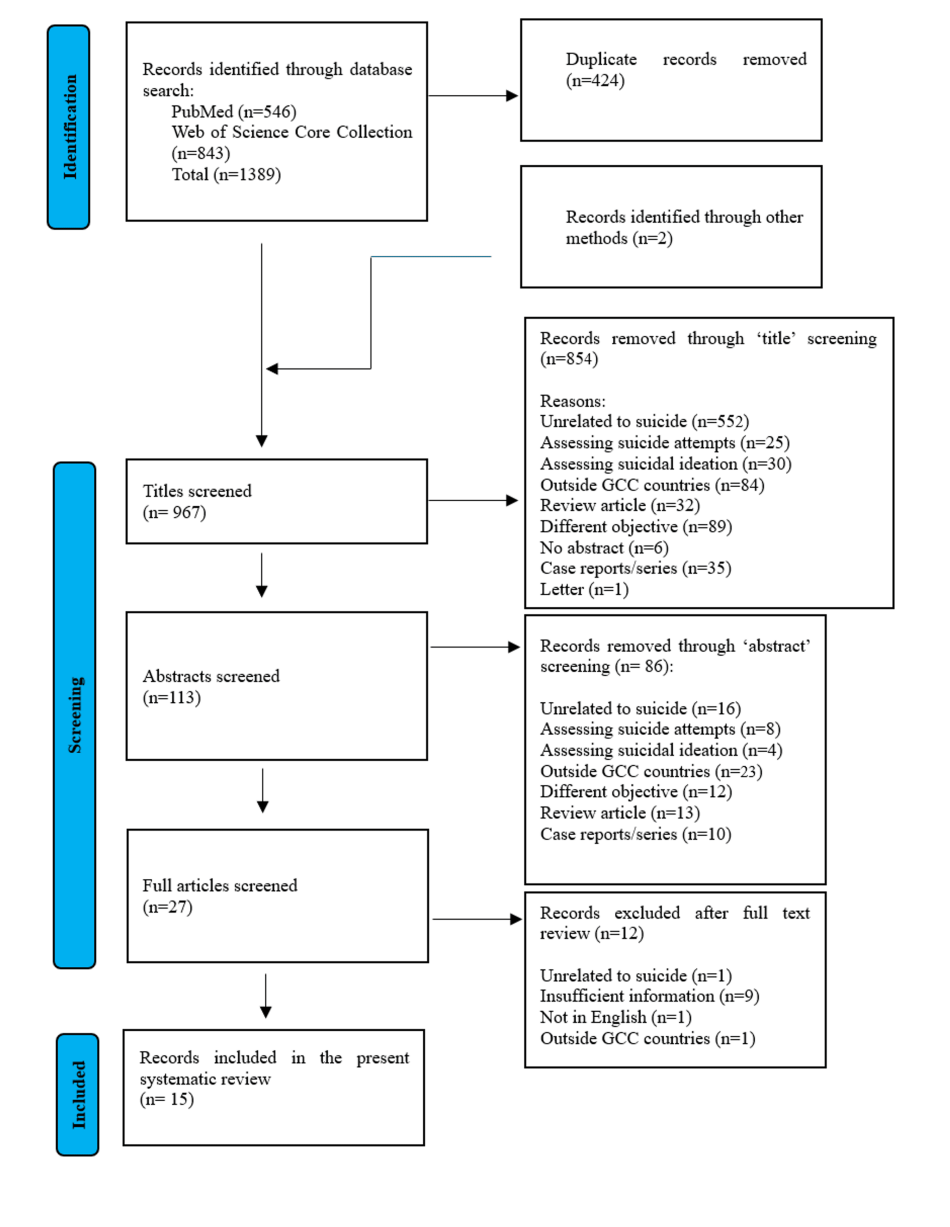
PRISMA flow chart mapping the number of records identified, screened, included and excluded, and the reasons for exclusions PRISMA: Preferred Reporting Items for Systematic reviews and Meta-Analyses

The identified records were imported into the Rayyan software [12] for removal of duplicates. The screening process consisted of three stages: initial title screening, abstract review, and full-text assessment, each conducted independently by two reviewers. A third reviewer resolved any discrepancies regarding the inclusion or exclusion of records. Records lacking abstracts, with restricted access, without original data, or with insufficient reported outcomes were excluded. Ultimately, 15 records (studies/articles) met the inclusion criteria and were incorporated into this review, as illustrated in the Preferred Reporting Items for Systematic reviews and Meta-Analyses (PRISMA) flowchart (Figure 1) [1-4,13-24]. After completing the screening process, data from the final relevant records were extracted by two independent reviewers and verified by a third reviewer. Details such as authorship, publication year, and study design are summarized in Table 2.

**Table 2:** Baseline characteristics of the studies included in the present review

Reference	Year of Publication	Country	Study period	Method
Al-Amin et al. [14]	2021	Qatar	2013 - 2014	A retrospective review of 37 suicides presented to the Emergency Department of Hamad Medical Corporation in Doha
Al-Waheeb et al. [1]	2020	Kuwait	2014 – 2018	A retrospective review of 297 suicides investigated at the General Department of Criminal Evidence
Issa et al. [15]	2016	Saudi Arabia	2012 - 2013	A retrospective review of 145 suicides examined at the Forensic Medicine Center in Dammam
Al-Waheeb and Al-Kandary [2]	2015	Kuwait	2003 – 2009	A retrospective review of 347 suicides investigated at the General Department of Criminal Evidence
Helaly et al. [16]	2015	Saudi Arabia	2008–2012	A retrospective review of 200 suicides examined at the Forensic Medicine Center in Jeddah
Madadin et al. [17]	2013	Saudi Arabia	2000 - 2003	A retrospective review of 126 suicides examined at the Forensic Medicine Center in Dammam
Abd-Elwahab Hassan et al. [3]	2013	Kuwait	2010 – 2012	A retrospective review of 118 suicidal hanging cases examined at the Institute of Forensic Medicine
Dervic et al. [18]	2011	United Arab Emirates	2003 - 2009	A retrospective review of 594 suicides investigated by the Dubai Police General Headquarters
Al Madni et al. [19]	2010	Saudi Arabia	2003–2007	A retrospective review of 133 suicidal hanging cases examined at the Forensic Medicine Center in Dammam
Benomran [20]	2009	United Arab Emirates	2002–2007	A retrospective review of 498 suicides investigated by the Dubai Police General Headquarters
Al Ansari and Ali [21]	2009	Bahrain	1996 – 2005	A questionnaire-guided interview of families of 29 Bahraini suicide cases
Al Ansari et al. [4]	2007	Bahrain	1995 – 2004	A retrospective review of 304 suicides investigated by the Directorate of Criminal Investigation at the Ministry of Interior
Koronfel [22]	2002	United Arab Emirates	1992 - 2000	A retrospective review of 362 suicides investigated by the Dubai Police General Headquarters
Elfawal [23]	1999	Saudi Arabia	1986 – 1995	A retrospective review of 221 suicides examined at the Forensic Medicine Center in Dammam
Elfawal and Awad [24]	1994	Saudi Arabia	1988 - 1992	A retrospective review of 59 suicidal hanging cases examined at the Forensic Medicine Center in Dammam

Results

Of the 967 records identified for screening after removal of the duplicates, 15 records met the inclusion criteria [1-4,14-24]. Six studies were conducted in Saudi Arabia [15-17,19,23,24], three in Kuwait [1-3], two in Bahrain [4,21], three in Dubai, United Arab Emirates [18,20,22], and one in Qatar [14]. Some overlap of data was observed in some of the retrospective studies conducted on data from the available databases in a few countries (Table 2). 

Demographics

Gender: Across all studies, male subjects were the predominant group.

In Dammam, Saudi Arabia, Issa et al. documented that male victims constituted 84.1% of suicides, while female victims constituted 15.9%, with a male-to-female ratio of 5.3:1 [15]. Elfawal and Awad found that out of 59 cases of suicidal hanging, most of the victims were male [24]. Al-Madni et al. reported that 86.46% of suicidal hanging victims were male with a rate six times higher than that of females, 13.54%, with yearly ratios reaching as high as 14:1 [19]. Madadin et al. found a nearly identical gender distribution when compared to the previous study from the same center, with men comprising 86.5% of cases, while women comprised 13.492% of the cases, yielding a male-to-female ratio of 6.4:1, with the highest ratio reaching 10:1 in 2000 [17]. Moreover, Elfawal reported that 82% of the victims were male (ratio 4.5:1) [23]. In the Western Region, Saudi Arabia, Helaly et al. reported that 72% of victims were male and 28% were female [16].

In Kuwait, Abd-Elwahab Hassan et al. reported a male predominance, with 73% of victims being male and 27% female [3]. From 2014 to 2018, Al-Waheeb et al. also reported a predominantly male distribution, with men accounting for 81% and women 19% of cases [1]. While in another study from 2003 to 2009, Al-Waheeb and Al-Kandary similarly reported that men constituted 70% of suicides, while women represented 30% [2]. The study also showed that more men died from suicide compared to women in all suicidal methods, except for jumping from a height [2].

In Dubai, United Arab Emirates, the study by Dervic et al. showed that among 594 suicide cases, 551 were male, while 43 were female [18]. The study reported a male-to-female suicide rate ratio of 4.1:1 [18]. Koronfel noted that 85% of the victims were male [22]. Koronfel also reported that the highest recorded annual male-to-female ratio was 5.75:1 in 1999 [22]. Furthermore, a male-to-female ratio of 7.7:1 among Muslims compared to 4.9:1 among non-Muslims was reported [22]. Benomran did not report a gender breakdown specifically for suicide victims [20].

In Bahrain, Al Ansari and Ali reported a marked male predominance, with 93.1% of victims being male, and 6.9% being female, resulting in a male-to-female ratio of 13.5:1 [21]. In another study, Al Ansari et al. similarly found that the male suicides exceeded that of females by a ratio of 6:1 [4].

In Qatar, the study by Al-Amin et al. showed that all of the suicide victims were male [14].

Age: The age distribution in all studies shows a higher prevalence among younger adults.

In Dammam, Saudi Arabia, Issa et al. reported that the largest proportion of victims (38.6%) were aged 21-30 years, followed by those aged 31-40 years, while a few cases occurred above the age of 60 [15]. Al-Madni et al. found that most suicidal hanging deaths involved adults aged 21-50 years (88.7%), with the fourth decade representing the highest concentration (36.09%); only three cases occurred in the 10-19 age group and two above age 60 [19]. They further reported that 66% of the female victims were in the fourth decade, and the rest were in the third decade [19]. There was no case aged lower than 10 years, and the number of cases in the age group over 60 was the lowest (1.5%) [19]. Madadin et al. similarly reported that approximately 88% of victims were in their third, fourth, and fifth decades of life, with the fourth decade accounting for 53.17% of all cases; 76.47% of the female victims were in their fourth decade [17]. Only two cases occurred in the seventh-decade age group, one case occurred in the second decade, and none were reported in individuals under the age of 10 [17]. Elfawal found that most victims were young adults, with 44.3% aged 30-39 years and 32.6% aged 20-29 years, while very few cases occurred in individuals under 20 years (1.8%) or over 60 years (3.2%) [23]. The youngest victim was 13 years old [23]. In another study, Elfawal and Awad found that 54% of victims were between 30-39 years and 23% between 20-29 years, with only one case reported in a child younger than 10 years [24]. In the Western Region, Saudi Arabia, Helaly et al. reported that 34% of victims were aged 31-40 years, and the second-most common age group was 21-30 years, with nearly three-quarters of suicides occurring between 21 and 40 years and far fewer cases below age 20 or above age 50 [16].

In Kuwait, Abd-Elwahab Hassan et al. reported that 87.3% of victims were between 21 and 50 years of age, with the highest proportion occurring in the third decade of life (approximately 43%) [3]. The study reported no victims under 10 years and only two victims older than 60 years [3]. Similarly, Al-Waheeb et al. identified in a study from 2014 to 2018 that the mean age was 34.05 years [1]. The oldest victim was 77 years old, while the youngest was 10 years old [1]. The majority of the victims were aged 19-35 years (60.6%) and 36-65 years (36%) [1]. In another study from 2003 to 2009 by Al-Waheeb and Al-Kandary, the age ranged from 17 to 62 years, with the 20-29 and 30-39 groups accounting for most hanging suicides (39% and 41%, respectively) [2]. The over-60 and under-19 age groups had the lowest number of suicides by hanging (2% and 0.5% respectively) [2]. The 30-39 group represented the largest proportion of suicides involving sharp-force injuries, poisoning, and firearms (50%, 49%, and 39%, respectively) [2]. No case of suicide by firearm was reported in the age group over 60 [2].

In Dubai, United Arab Emirates, Dervic et al. reported that 59.6% of suicide victims were older than 30 years, while 39.4% were aged 30 years or younger [18]. Only three victims (0.5%) were younger than 18, and age information was missing in six cases (1%) [18]. Koronfel showed that nearly 81.5% of suicide victims were aged 21-40 years [22]. Among these cases, 29% occurred in individuals aged 26-30 years, whereas the lowest incidence (2.7%) was observed among those older than 50 years [22]. Benomran did not provide age data specific to the suicide subgroup [20].

In Bahrain, Al Ansari and Ali examined 29 Bahraini suicide victims between 1996 and 2005, reporting that 48.3% were younger than 30 years and 44.8% were aged 30-44 years; no victims were older than 60 [21]. Furthermore, in a study from 1995 to 2004, Al Ansari et al. noted a mean age of 34 years, with the highest proportion of suicides occurring among those aged 30-34 years (25%), followed by individuals aged 25-29 years (23%) [4]. Only three Bahraini male victims died by suicide below the age of 20, and only one non-Bahraini male died above the age of 60. The mean age for Bahraini nationals was 31 years compared to 34 years for non-Bahrainis [4].

In Qatar, Al-Amin et al. reported that all suicide victims were in their mid-30s [14].

Nationality: Expatriates constituted a substantial proportion of the victims.

In Dammam, Saudi Arabia, Issa et al. found that 80% of victims were non-Saudis, with Indians comprising the largest proportion (44.8%), followed by Nepalese, Filipinos, Sri Lankans, Sudanese, Pakistanis, Syrians, and smaller numbers of additional nationalities; Saudis represented 20% of cases [15]. Al-Madni et al. also reported that foreign nationals constituted the majority at 84.2%, with Indians again forming the largest group (47.4%); Saudis accounted for 15.8% of victims, and the remainder represented 11 additional nationalities, each contributing fewer than eight cases-including Bangladeshis, Filipinos, Sri Lankans, Indonesians, Nepalese, Yemenis, Sudanese, Iraqis, British, Turkish, Afghani, and Qatari individuals [19]. Elfawal and Awad reported an even higher proportion of expatriates (87%), with Indians accounting for more than half of all cases (54%), and smaller proportions from Thailand, Sri Lanka, Bangladesh, Philippines, Pakistan, Indonesia, Turkey, and two British nationals; Saudis represented only 13% of victims [24]. Madadin et al. found that 84.92% of cases involved foreign nationals. Furthermore, the majority were Indians (42.85%), followed by Saudi nationals (15.07%), Nepalese (8.73%), Bangladeshis (7.14%), Filipinos (7.14%), Indonesians (6.34%), Sri Lankans (4.76%), Pakistanis (3.17%), and one case of unknown nationality; four other nationalities comprised only 3.96% of cases, each with only one case: Yemeni, Sudanese, British, and Afghani [17]. Elfawal also documented that foreign nationals constituted 77% of all suicides, led by South Asians, Indians comprising 43% of total deaths, followed by East Asians and various other expatriate groups; Saudis made up only 23% of victims [23]. In the Western Region of Saudi Arabia, Helaly et al. found that Indians were the most represented nationality (23%), followed by Saudis (18.5%), Ethiopians (11%), Filipinos (7.5%), Yemenis (6%), and Afghans (5%) [16]. Other expatriate groups formed the rest of the minor proportion of cases [16].

In Kuwait, Abd-Elwahab Hassan et al. reported that only 5.9% of victims were Kuwaiti nationals, while 94.1% were non-Kuwaiti expatriates. Among these, Indians constituted the largest group (54.8%), followed by Nepalese (13.6%), Ethiopians (10%), and additional victims from Bangladesh, Egypt, Sri Lanka, Indonesia, Syria, Pakistan, Afghanistan, the United States, the Philippines, and Yemen [3]. In a study conducted between 2014 and 2018, Al-Waheeb et al. similarly found that Indians constituted the majority of cases (60.2%), followed by Bangladeshis (8.4%), Kuwaitis (7.4%), Sri Lankans (5.4%), and Nepalese (4.7%) [1]. From 2003 to 2009, Al-Waheeb and Al-Kandary noted that 87% of victims were non-Kuwaiti and 13% Kuwaiti nationals [2].

In Dubai, United Arab Emirates, Dervic et al. reported that Asia accounted for 93.8% of expatriate suicides, with India accounting for 78.6% and other Asian countries for 15.2% of the cases [18]. People from other countries committed the remaining 6.2% of expatriate suicides [18]. Koronfel noted that 94% of victims were expatriates [22]. Indians constituted 79% of the cases [22]. Benomran did not report the nationalities of the victims specific to the suicide subgroup [20].

In Bahrain, Al Ansari et al. reported that suicide among Bahrainis was uncommon, with a mean suicide rate of 0.6 per 100,000, compared to 12.6 per 100,000 among non-Bahrainis [4]. Among expatriates, Indians had notably high suicide rates, reaching 17.7 per 100,000 in 2001 [4].

In Qatar, the study by Al-Amin et al. showed that only one case was from Qatar, two were Arabs, and the rest were expatriates [14].

Marital status: In Saudi Arabia, Elfawal reported that 41% of male victims were married, while 65% of female victims were married [23]. Similarly, in Kuwait, from 2003 to 2009, Al-Waheeb and Al-Kandary reported similar findings, noting that 49% of victims were married and 51% were non-married [2]. In Dubai, United Arab Emirates, Dervic et al. noted that 48.6% of victims were single, 38.4% were married, and 3.4% were widowed or divorced, while marital status was unknown in 9.6% of cases [18]. In Bahrain, Al Ansari and Ali showed that 51.7% of victims were single, 37.9% were married, and 10.3% were widowed or divorced [21]. Other studies did not report marital-status information [1,3,4,14-17,19,20,22,24].

Methods of Suicide

Across all the studies in this review where the method of suicide data was provided, hanging was the most common method of suicide. However, it should be noted that three studies focused only on hanging [3,19,24].

In Dammam, Saudi Arabia, Issa et al. identified hanging as the predominant method of suicide (55.1%), followed by poisoning (10.3%), primarily involving organophosphates [15]. Less common methods included firearm injuries and stab wounds (4.1%), cutthroat injuries (2.8%), and falling from a height and self-burning (1.4%) [15]. Similarly, Madadin et al. reported that hanging accounted for 89.68% of the cases [17]. Other methods included firearms (5.56%), falling from a height (1.59%), poisoning (1.59%), throat-cutting (0.79%), and drowning (0.79%) [17]. Elfawal observed hanging as the most prevalent method (63%), dominating nearly all nationalities and both genders [23]. Jumping from a height ranked second at 12% and occurred more frequently among males [23]. The frequency of jumping from a height was also variable among subgroups. To illustrate, it accounted for 14% of East Asian suicide cases compared to 9% in South Asians [23]. Firearm suicides constituted 9% of the cases and were concentrated almost entirely among Saudis due to greater access to weapons; among Saudis, firearm suicides occurred nearly as frequently as hanging [23]. Poisoning accounted for 6% of suicides and included overdoses, ingestion of insecticides, and carbon monoxide exposure [23]. Other suicide methods were rare in the study group; these included suicide by fire, representing 7% of South Asian suicides; self-cutting/stabbing, representing 14% of East Asian cases; and drowning [23]. Al-Madni et al. reported exclusively on suicidal hanging cases, noting that these accounted for 83.1% of all suicides autopsied between 2003 and 2007 [19]. Similarly, Elfawal and Awad focused exclusively on hanging deaths, reporting 59 suicidal hangings [24]. In the Western Region, Saudi Arabia, Helaly et al. also reported that hanging was the predominant method of suicide, representing 72% of all cases and constituting the leading method across both genders and the majority of nationalities [16]. Among male patients, 87% of suicides resulted from hanging, compared to 34% among females. Hanging accounted for 82% of suicides among Indian nationals, while 57% of Saudi suicides employed this method [16]. Other methods included falling from a height (8.5%), burning (5%), firearms (5%), drug overdose (4.5%), drowning (4%), and cut-throat (less than 1%) [16]. Across nearly all age groups below 50 years, hanging represented between 69% and 77% of suicides, decreasing to 50% among those older than 50 [16].

In Kuwait, Abd-Elwahab Hassan et al. reported exclusively on suicidal hanging cases between 2010 and 2012 [3]. Al-Waheeb and Al-Kandary examined suicide patterns in the period from 2003 to 2009 [2]. Hanging remained the most common suicide method, accounting for 60% of cases. Suicide by using sharp objects represented the second most frequent method (17%), followed by poisoning (14%), and firearms (5%) [2]. Falling from a height was the least common suicide method, accounting for 4% of the sample, and it was exclusive to non-Kuwaitis. Male victims represented the majority of suicide cases in all suicide methods, except in falling from heights, where female victims represented 69% of the cases. Also, all suicide methods were more frequent in non-Kuwaitis, except firearms, where Kuwaitis made up 67% of the cases [2]. Al-Waheeb et al. published a study in 2020 examining 297 cases of suicide that took place between 2014 and 2018 [1]. The study showed a predominance of hanging as the method of choice (90.6%), followed by falling from a height (7.1%), gunshots (1.7%), and suicide by burning, which was the least common method (0.7%) [1].

In Dubai, United Arab Emirates, Benomran found hanging to be the predominant method, accounting for 80% of all suicides [20]. Falling from a height represented 5.7% of cases, and drowning 2.8%, while burns accounted for 2.6% of deaths. Ingestion of corrosive substances and insecticides accounted for 1.4% each [20]. Koronfel reported that hanging was the most common method, accounting for 75% of cases, occurring in 78.8% of males and 54.5% of females [22]. It was also the most frequent method among Indian expatriates (82.8%), followed by citizens (71.4%), and other expatriates (36.4%). Jumping from a height represented 9.7% of cases, occurring in 7.8% of males and 20% of females, and was more common among other expatriates (34.5%) than citizens (9.5%) or Indians (4.9%). Poisoning accounted for 7.2% of suicides, occurring in 5.5% of males and 16.4% of females. The most common substances used in the self-poisoning cases were corrosives (23%), followed by pesticides (19.2%) and carbon monoxide (11.5%). Other substances included paracetamol, barbiturates, tricyclic antidepressants, organic solvents, and plant poisons, each accounting for 7.7%, while diazepam and dextropropoxyphene each accounted for 3.8% [22].

In Bahrain, Al Ansari et al. documented that hanging was the overwhelmingly predominant method, accounting for 92.8% of all suicides [4]. Drowning constituted 3.8% of cases, followed by burning (1.3%) and stabbing injuries (1.3%). Carbon monoxide poisoning accounted for 0.7% of suicides, while jumping from a height represented 0.3%. Only one case involved the use of a firearm [4].

In Qatar, Al-Amin et al. reported that among the 37 completed suicides occurring between 2011 and 2012, 95% resulted from hanging using a rope, and the study did not document any other methods among these fatal cases [14].

Risk Factors

In Dammam, Saudi Arabia, Issa et al. identified several clinical and psychosocial contributors to suicide [15]. Toxicology screening showed a high prevalence of substance use, with ethanol detected in 62% of the cases, amphetamines in 13.8%, cannabinoids in 12.4%, and opioids in 10.4%; polysubstance use was common, occurring in 79% of substance-positive cases. Psychological profiling demonstrated that female victims had higher measured levels of stress, depression, and previous self-harm than male victims, while unemployed individuals similarly exhibited higher stress levels and increased histories of self-harm compared with employed victims. Strong positive correlations were reported between depression and previous self-harm. Financial problems were the most frequently reported factor among non-Saudis (47%) compared with Saudis (26%), whereas psychological illness was most commonly reported among Saudi nationals (47% vs. 5%), followed by family disputes [15]. Al Madni et al. noted a history of psychological illness in 14.28% of the cases, stressful family problems in 6% of victims, and only one individual with a previous suicide attempt [19]. Toxicological analysis identified ethyl alcohol in 6.76% of cases, amphetamine in 3%, and cannabinoids in 2.25%. The study also reported that 75% of victims were male laborers, 11.27% were female housemaids, and 9.77% were unemployed [19]. Madadin et al. reported multiple psychosocial and clinical contributors [17]. Family troubles were documented in 5.5% of cases, psychological illness in 13.49%, and other identified stressors, including financial problems, work-related stress, recent arrival in the country, and chronic illness, in 8.73%. Four victims had a documented history of previous suicide attempts. Toxicological analysis showed ethyl alcohol in 5.55% of cases, amphetamine in 3.96%, and cannabinoids in 1.59% [17]. In another study, Elfawal and Awad identified psychological and social stressors among victims, reporting a history of depressive symptoms in approximately 39% of cases and prior suicide attempts in three individuals [24]. Family-related problems were documented in 15% of expatriate victims. The study emphasized that many suicides occurred among young male expatriates working in low-income labor sectors (85%) [24].

In Dubai, United Arab Emirates, Koronfel reported that information on prior psychological illness or trauma was available in 9.7% of cases [22]. Among these, 12 individuals were receiving treatment for depression at the time of death. Financial or occupational stressors were identified in nine cases, including recent job loss or debt. Substance abuse was documented in three victims. Terminal illness was reported in two cases, both involving advanced cancer. A recent divorce was documented in two cases, while family disputes preceded suicide in four cases. Additional circumstances included the death of a spouse, an illegitimate pregnancy, and recent imprisonment, each reported in one case. In three cases (0.8%), homicide or attempted homicide of a spouse and/or child preceded the suicide. Evidence of previous suicide attempts was identified in six cases (1.65%). 28% of the cases tested positive for alcohol. Furthermore, sublethal levels of drugs were observed in 6.4% of cases, which included cannabis, opiates, and barbiturates; one case each. Three cases of glue sniffing (toluene), five cases of diazepines, and one case of mixed diazepam and barbiturate abuse were reported. In 25% of the cases, drug abuse was associated with alcohol intoxication prior to the incident [22].

In Bahrain, Al Ansari and Ali conducted a study, during which clinical and psychosocial risk factors were extensively documented [21]. Psychiatric illness was highly prevalent, with 51.7% of victims having a diagnosed mental disorder, including schizophrenia (24.1%), mood disorders (13.9%), personality disorders (3.4%), and substance-use disorders (10.3%). Notably, more than half (53%) of those with psychiatric conditions had been ill for over five years. A history of alcohol or drug use was present in 31.0% of cases, though none were registered for formal treatment. Prior suicide attempts were documented in 10.3%, and 10.3% had a family history of suicide in first-degree relatives. Psychosocial stressors were widespread and included family problems (44.8%), financial difficulties (27.6%), and relationship problems (24.1%). Legal and criminal history was also prominent, with 31.0% of victims being involved in crime or legal disputes [21]. In another study, Al Ansari et al. identified financial or domestic problems, receipt of distressing news from the country of origin, and relationship difficulties as the circumstances preceding suicide [4].

Other studies did not provide victim-specific risk-factor data [1-3,14,16,18,20,23].

Discussion

This systematic review provides an analysis of suicide patterns in GCC countries. In GCC countries, suicide predominantly affects males, who are consistently overrepresented across studies [1-4,14-24]. The male predominance was also observed in studies conducted in non-GCC Muslim countries like Egypt and Iran, as well as Western countries [25-27]. The gender disparity may be linked to greater determination to commit suicide among men, while women may be more likely to engage in less severe suicidal behavior manifestations as a form of distress communication rather than a direct determination to die [28]. Not all studies included in the present review commented on the religion of the victims. Nevertheless, it is reported that non-Muslims exhibit higher suicide rates than Muslims, which may be influenced by religious and cultural factors, such as the Islamic prohibition of suicide and the emphasis on patience in adversity [29]. Expatriates, especially manual laborers and domestic workers, are at a higher risk of suicide than nationals, likely due to increased stress, limited social support, and challenging work or living conditions [1-4,14-19,22-24].

Furthermore, the findings of this review are consistent with non-GCC literature regarding the age distribution of suicide cases. An Egyptian study reported that the highest proportion of suicides occurred among individuals aged 21-30 years (32.2%), followed by those aged 31-40 years (25.5%) [26]. Similarly, a study from Iran demonstrated a comparable pattern, with a peak incidence among individuals aged 20-29 years [25]. This concentration of cases within the economically productive age groups may reflect increased vulnerability related to employment pressures, financial responsibilities, and psychosocial stressors, as well as the predominance of this age group within expatriate workforces in the GCC region [4,22].

Considerable heterogeneity was observed in the reporting of marital status across studies, with both married and non-married individuals comprising substantial proportions of suicide victims, when reported. This variability suggests that marital status may not serve as a consistent protective or risk factor within the GCC context. Globally, marriage has traditionally been regarded as protective against suicide through enhanced social integration and emotional support. However, a large meta-analysis by Kyung-Sook et al. demonstrated that although non-married individuals had a higher overall suicide risk than married individuals, this association was strongly moderated by age, gender, and cultural context [30]. Evidence from Iran adds further to this complexity [31]. An Iranian population-based study reported higher crude suicide fatality rates among married individuals; however, this association disappeared after age adjustment, indicating that age distribution rather than marital status itself accounted for the observed difference [31]. In contrast, widowed and divorced individuals remained at consistently higher risk, suggesting that marital dissolution may represent a more robust risk factor than marital status alone [31].

Hanging is the most prevalent method of suicide in GCC countries [1-4,14-24], likely due to its accessibility and lack of requirement for specialized resources. This trend is also observed in Iran, where hanging accounts for the majority of suicides among both genders [25]. By contrast, self-poisoning is the preferred method in Egypt and India, often involving easily accessible pesticides and substances such as aluminium phosphide [1,26]. In the United States, firearms are the leading method, reflecting their widespread availability and minimal restrictions [2,27]. These differences underscore the influence of cultural, regulatory, and environmental factors on the choice of suicide method across regions.

Several limitations should be acknowledged. For example, the inclusion of studies with varying methodological quality may restrict the validity of cross-study comparisons, as formal assessments of quality and bias were not performed. Suicide cases may also be underestimated due to misclassification as non-suicidal deaths such as accidental deaths. Additionally, the limited data from Qatar and Oman constrain the generalizability of findings across the entire GCC region. In fact, there were no studies reported from Oman. The exclusion of a substantial number of articles may have reduced the comprehensiveness of the review, and some included studies did not report their own limitations, which could have impacted the overall conclusions.

To address suicide in the GCC, several measures should be considered. These include increasing mental health literacy among both nationals and expatriates, with a focus on risk factors such as depression, substance abuse, and previous suicide attempts [32]. Efforts should also be made to raise awareness of available mental health resources and encourage help-seeking behaviors. Screening for depression and suicide risk, both prior to employment and during regular health check-ups, may facilitate early identification and intervention [33]. Furthermore, training non-psychiatrist healthcare professionals, including primary care physicians, to recognize and respond to suicide risk could be particularly impactful, given their frequent contact with at-risk populations [34].

## Conclusions

Suicide in GCC countries is influenced by a range of factors, including demographic characteristics, expatriate status, mental health conditions, and socioeconomic challenges. Young male expatriates are particularly vulnerable due to financial pressures, domestic difficulties, and limited social support. Despite these concerning trends, few targeted interventions or prevention strategies exist, and regional awareness of suicide and its risk factors remains low. Addressing the suicide burden in the GCC region necessitates culturally sensitive approaches and the development of region-specific policies.
